# Supplementary figures and images for: An optimized live imaging and multiple cell layer growth analysis approach using Arabidopsis sepals
Source: Front Plant Sci. 2024 Sep 3;15:1449195. doi: 10.3389/fpls.2024.1449195 (PMC11405221; doi:10.3389/fpls.2024.1449195)

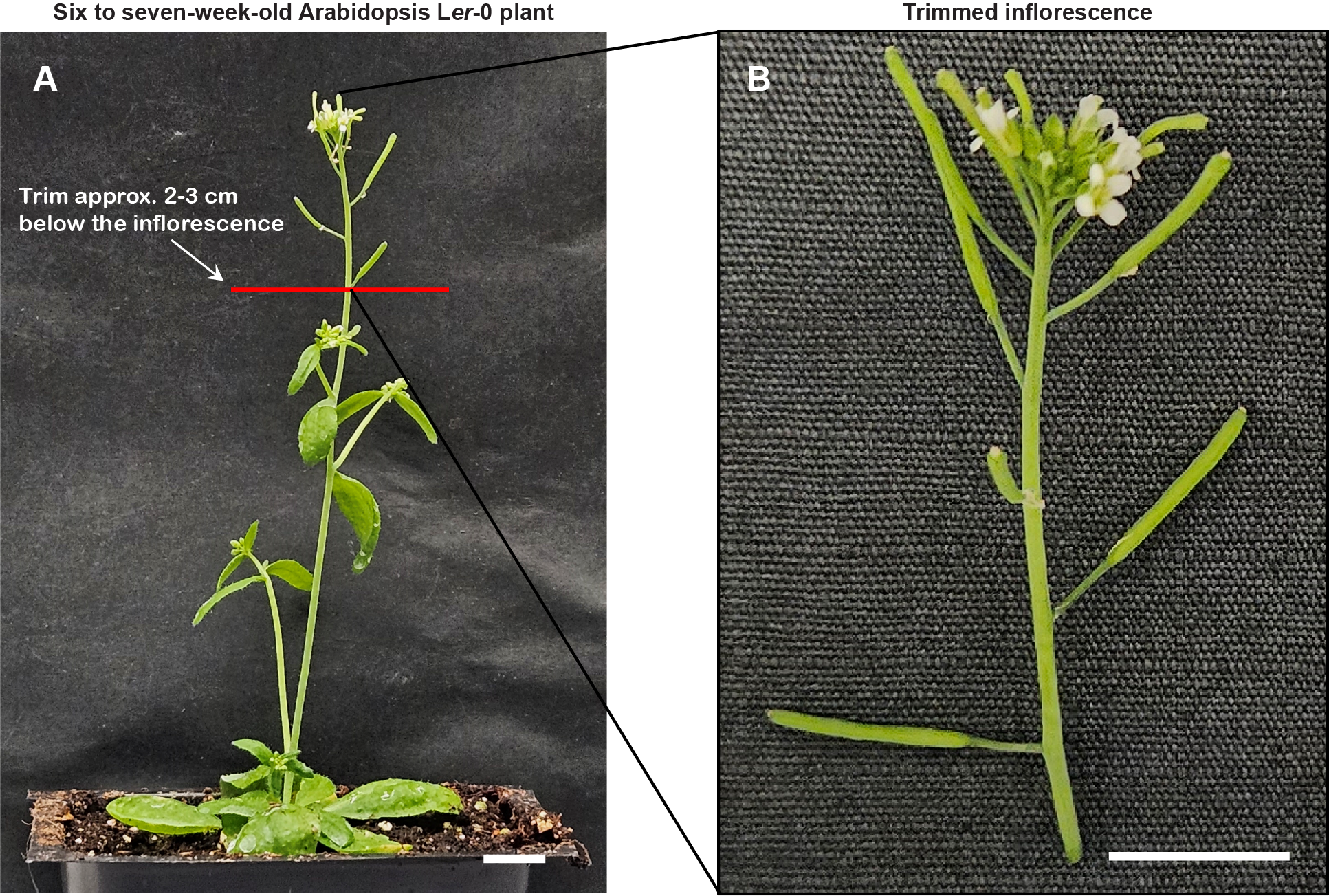

Supplement: Supplementary Figure 1 — Trimmed inflorescence for dissection, associated with Figure 1 . (A) Whole plant image of six to seven-week-old Arabidopsis Ler-0 accession plant grown in long-day conditions (16-hr light/8-hr dark). (B) Inflorescence post-trimming, which has been cut approximately 2-3 cm below the inflorescence apex (red line) for dissection. Scale bar, 1 cm. [file Image1.tif]

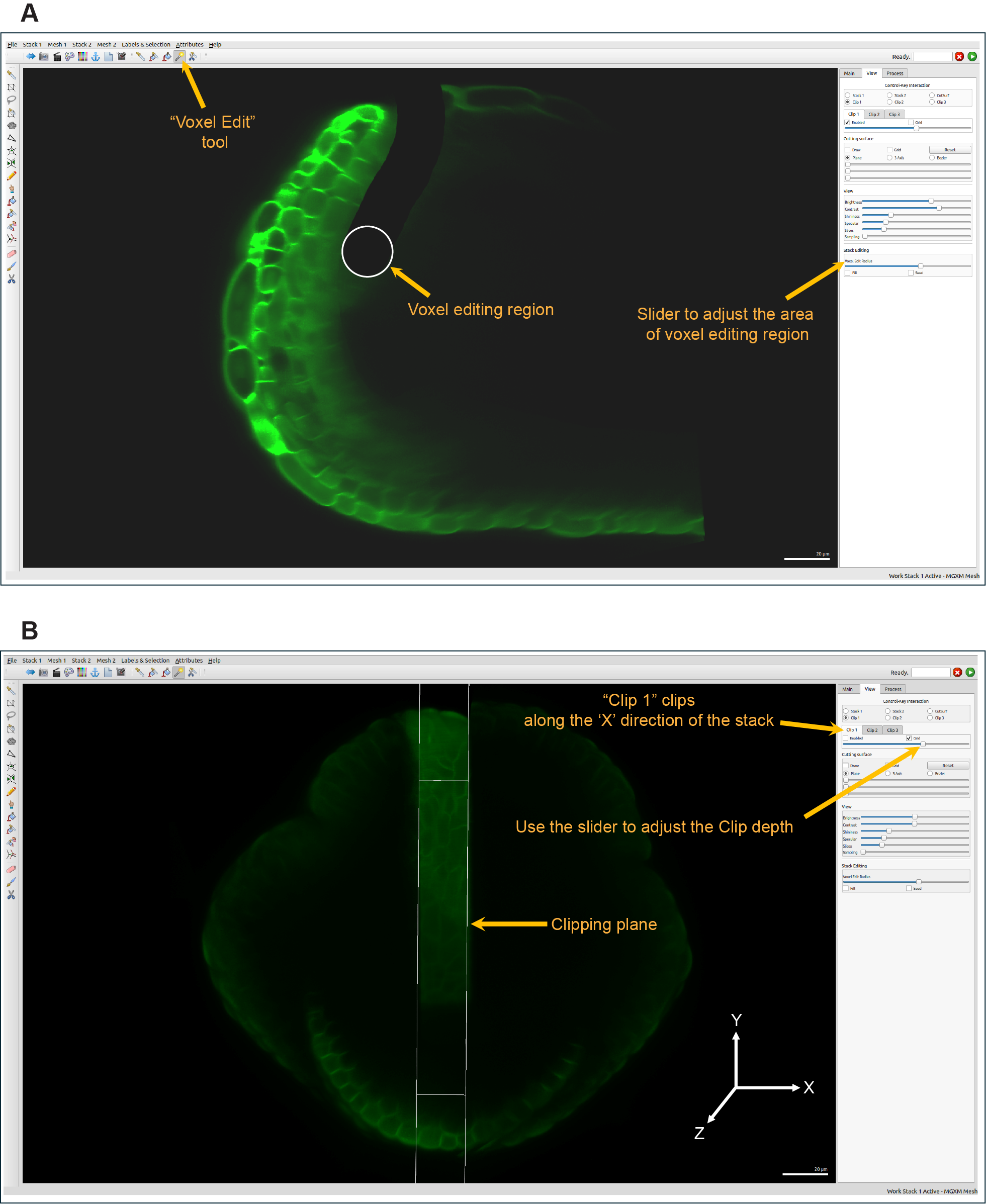

Supplement: Supplementary Figure 2 — The ‘Voxel Edit’ tool and ‘Clipping plane’ on the MorphographX window, associated with Figure 3 . (A) The panel shows the location of the “Voxel Edit” tool and the slider under the ‘View’ tab to adjust the area for voxel editing. One can also change the editing area by using the mouse scrolling wheel to zoom in and out. Pressing and holding the Alt-key activates the tool (a white circle will appear), allowing for voxel deletion by left-clicking the mouse. Note, for “Voxel Edit” option to be available, the image needs to be in the work stack (Process/Stack/MultiStack/Copy Main to Work Stack). (B) Panel shows the clipping plane. In this case, “Clip 1”, which is parallel to the Z-direction and clips along the X-direction of the stack, is shown. The slider allows for adjusting the depth (thickness) of the Clipping plane. [file Image2.tif]

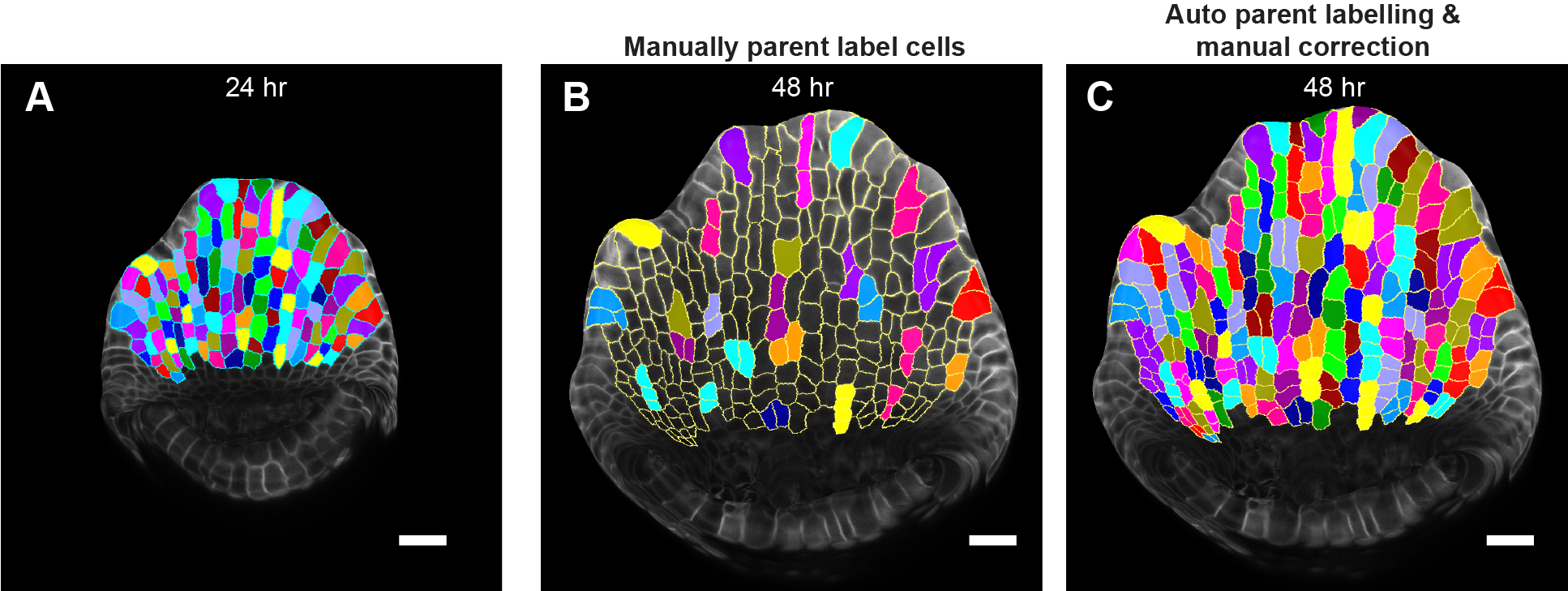

Supplement: Supplementary Figure 3 — Semi-automatic parent tracking, associated with Figure 5 . (A) MorphoGraphX generated mesh for the inner epidermis at 24-hour time point showing segmented cells. Colors represent individual cells. (B) Inner epidermis mesh at 48-hour time point, wherein segmented cells are being parent tracked to cells in 24-hour time point (panel A). Colors correspond to parent cells in the 24- hour time point mesh. Note that manually parent labelled cells are distributed across the mesh, which greatly aids automating parent labelling. (C) Cells in 48-hour time point parent labelled using automatic parent labeling, followed by manual correction. Scale bar, 20 µm. [file Image3.tif]
